# Nothing to lose: a grounded theory study of patients’ and healthcare professionals’ perspectives of being involved in the consent process for oncology trials with non-curative intent

**DOI:** 10.1186/s12904-020-00661-7

**Published:** 2020-10-30

**Authors:** Mary Murphy, Eilís McCaughan, Matthew A Carson, Monica Donovan, Richard H Wilson, Donna Fitzsimons

**Affiliations:** 1 Resuscitation Services, Elliott Dynes Building, Royal Victoria Hospital, Belfast Health and Social Care Trust, Belfast, UK; 2 School of Nursing and Midwifery, Institute of Nursing and Health Research, Ulster University, Coleraine, UK; 3 School of Nursing and Midwifery, Medical Biology Centre, Queen’s University Belfast, Belfast, UK; 4 Institute of Cancer Sciences, University of Glasgow, Glasgow, UK

**Keywords:** Neoplasms, Cancer, Clinical trial, Decision-making, Consent, Grounded theory

## Abstract

**Background:**

Clinical cancer research trials may offer little or no direct clinical benefit to participants where a cure is no longer possible. As such, the decision-making and consent process for patient participation is often challenging.

**Aim:**

To gain understanding of how patients make decisions regarding clinical trial participation, from the perspective of both the patient and healthcare professionals involved.

**Methods:**

In-depth, face to face interviews using a grounded theory approach. This study was conducted in a regional Cancer Centre in the United Kingdom. Of the 36 interviews, 16 were conducted with patients with cancer that had non-curative intent and 18 with healthcare professionals involved in the consent process.

**Results:**

‘Nothing to lose’ was identified as the core category that underpinned all other data within the study. This highlighted the desperation articulated by participants, who asserted trial participation was the ‘only hope in the room’. The decision regarding participation was taken within a ‘trusting relationship’ that was important to both patients and professionals. Both were united in their ‘fight against cancer’. These two categories are critical in understanding the decision-making/consent process and are supported by other themes presented in the theoretical model.

**Conclusion:**

This study presents an important insight into the complex and ethically contentious situation of consent in clinical trials that have non-curative intent. It confirms that patients with limited options trust their doctor and frequently hold unrealistic hopes for personal benefit. It highlights a need for further research to develop a more robust and context appropriate consent process.

## Background

Virtually all clinical research is contingent on informed consent [1–3]; which provides potential research participants with sufficient information, including anticipated benefits and potential risks of the study and any discomfort it may entail. This process is crucial, allowing patients to make a voluntary and autonomous decision as to whether participation is right for them [4]. Similarly, it is imperative participants understand the uncertainty or clinical equipoise involved, with alternative opportunities made fully available to them [5] - such as supportive care within a palliative context [6]. As early phase trials involve patients nearing the end of life and often promise little clinical benefit, [7] decision making presents a highly complex and ethically challenging situation [8].

The quality and validity of informed consent is critical, but previous research asserts that understanding can be limited and patients often confuse trial care with standard care [9–12]. Many factors have been identified as impacting on patients’ understanding, such as literacy [13], readability, complexity of consent forms and quality of explanations by the research team [14]. The patients’ state of mind may also be a factor, with some sources asserting patients in this situation are often motivated by hope of personal medical benefit [15–17], holding overly optimistic expectations of this benefit [18]. This has raised concerns of ‘therapeutic misconception’ [19], whilst others argue it is “therapeutic optimism” [20–22]. Contrastingly, it has also been argued that patients with an incurable disease nearing the end of life are too ill and therefore too vulnerable to take part in research [23]. It is hugely challenging to square off these differing perspectives; indeed the absence of an established evidence base in many areas of palliative care medicine has been partially attributed to the ethical challenges inherent in research within this population - including problems with consent [24].

Whilst there have been many advances made in relation to improving the informed consent process for clinical trial participation, such as the development of decisional aids [25], there is insufficient evidence to suggest these are effective in improving the decision-making process [26]. Other developments, such as improved training for healthcare professionals involved in research, have demonstrated beneficial outcomes [27]. Despite this, various sources assert that more research with life limited patients is needed to facilitate much needed improvements in the quality of their care [24, 28]. However, an improved understanding of patients’ decision-making in relation to informed consent is necessary to facilitate this. This study aims to address this gap in our knowledge of the consent process through the use of in-depth interviews, with both cancer patients who had been offered participation in a clinical trail and healthcare professionals who were involved in the consent/recruitment process.

## Methods

### Study aim

To gain understanding of how patients make decisions regarding clinical trial participation, from the perspective of both the patient and healthcare professionals involved.

### Study design

The study was conducted in a regional Cancer Centre in the UK. In-depth, face to face interviews were conducted using a grounded theory approach, as this is a common and appropriate methodology to explore how people experience transition in their lives [29]. It is particularly appropriate for this study given its focus on people’s behaviour, concerns [30] and its theoretical explanation for how problems are managed [31]. Furthermore, as this topic area is understudied at present, it is particularly well suited for a grounded theory approach which is recognized as having capacity to add breadth and depth to new areas of investigation [32].

### Participants

Eligibility criteria for patients: those previously invited to participate in a clinical trial, non-curative solid tumours or non-curative haematological malignancy; with > 12 weeks and < 5 years projected survival. Healthcare professionals were those identified by the patient as contributing to their consent related decision-making or trial recruitment, including both research nurses and doctors. Exclusion criteria for patients: less than 18 years of age, those being cared for by members of the research team and having a poor understanding of spoken English. All participants provided written informed consent.

### Recruitment

Eligible patients were identified by their Oncologist, who briefly outlined the study to them. If there was an expression of interest, the patient was given the participant information document and was asked for verbal agreement to be contacted by the researcher. All patients who were contacted agreed to participate. For patients, a convenience sample approach was used, whilst healthcare professionals were recruited via theoretical sampling after analysing patient data - as patients often identified oncologists or nurses that had an influence on their decision making. Professionals were first approached by email, giving them an outline of the study - if interested, full written consent was provided prior to them being interviewed.

### Interviews

All interviews were conducted by one researcher (MM), a female healthcare worker who was studying for a PhD at the time of data collection. This researcher was trained in interviewing skills and grounded theory, and had no prior relationship with patient participants. Given the researcher's clinical background, a conscious effort was made to remain open-minded during data collection and analysis (facilitated through use of multiple coders, field notes and memos). A formal interview guide was not used, as this would be at odds with the emergent nature of grounded theory – instead each interview began with a general request: ‘tell me about when you were first diagnosed with cancer’. Follow up questions varied based on the response of interviewees, though common prompts included: do you remember how you felt when you received your diagnosis/prognosis? When was a clinical trial first mentioned? How did you make your decision about participation?

Patient interviews were conducted as soon as reasonably possible after consent for the clinical trial had been given or refused, and prior to patients’ first disease reassessment (6–12 weeks). On one occasion a spouse was present during the interview as the patient had hearing difficulties. Most participants were interviewed once, though two patients were interviewed a second time. These follow up interviews were undertaken to give the interviewer a better understanding of some of the participants' previous points, rather than being an effort to generate new data. Interviews were conducted in a place of the participants’ choice, including the patient’s home (*n* = 16) and on three occasions in a private hospital room.

Healthcare professionals were interviewed in a quiet room on hospital premises. The researcher’s prior relationship with healthcare professionals was minimal, being limited to an awareness of each other’s professional capacity. On average, interviews lasted approximately 60 min (40–80 min).

### Data analysis

Interviews were digitally recorded (audio) and transcribed as soon as possible after the interview took place. This is in keeping with the constant comparative method, which enabled initial analysis to be completed prior to the next interview [33]. Field notes were made after each interview. Memos were also written and coded. Data collection was concluded when all members of the team were satisfied that emerging themes had been fully explored. Analysis was performed using the grounded theory techniques of open coding (*n* = 20), selective coding (*n* = 9) and theoretical coding (*n* = 3) [34]. Initial transcripts were coded by MM, DF and EMcC, after which identified codes were compared. Coding was completed by hand, as initial use of NVivo resulted in the researcher feeling too ‘distanced’ from the data. Developing codes were reviewed and refined by the study investigators as analysis progressed.

## Results

Demographics and clinical characteristics of the patient sample are displayed in Table 1. The sample was predominantly male (11/16), with a mean age of 57.6 years. There was a range of different cancer types, though patients were mostly participating in a Stage III trial. Updated analysis confirms that they were interviewed relatively close to death (7–64 weeks). In terms of the healthcare professional sample, this was a predominantly female population (10/18) – comprised of ten Oncologists and eight Clinical Research Nurses.

**Table 1:** Patient characteristics

Participant	Gender	Cancer Diagnosis	Trial Phase	Accept trial?	Time from initial interview to death
P01	F	Breast	I	Yes	10 weeks
P02	M	Oesophagus	III	Yes	16 months
P03	F	Pancreatic	III	No	7 weeks
P04	M	Prostate	III	Yes	Alive
P05	F	Breast	I	Yes	3 months
P06	M	Prostate	III	Yes	29 months
P07	M	Prostate	III	No	3 months
P08	M	Prostate	III	Yes	Alive
P09	M	Colorectal	II	Yes	16 months
P10	M	Pancreatic	III	Yes	17 months
P11	M	Pancreatic	III	Yes	11 months
P12	F	Myeloma	III	Yes	Alive
P13	M	Pancreatic	III	No	6 months
P14	M	Colorectal	II	Yes	5 months
P15	F	Breast	I	Yes	9 months
P16	M	Liver	III	Yes	14 months

Table legend: *P* = patient

### Category 1 (core category) – NOTHING TO LOSE

**Fig. 1:**
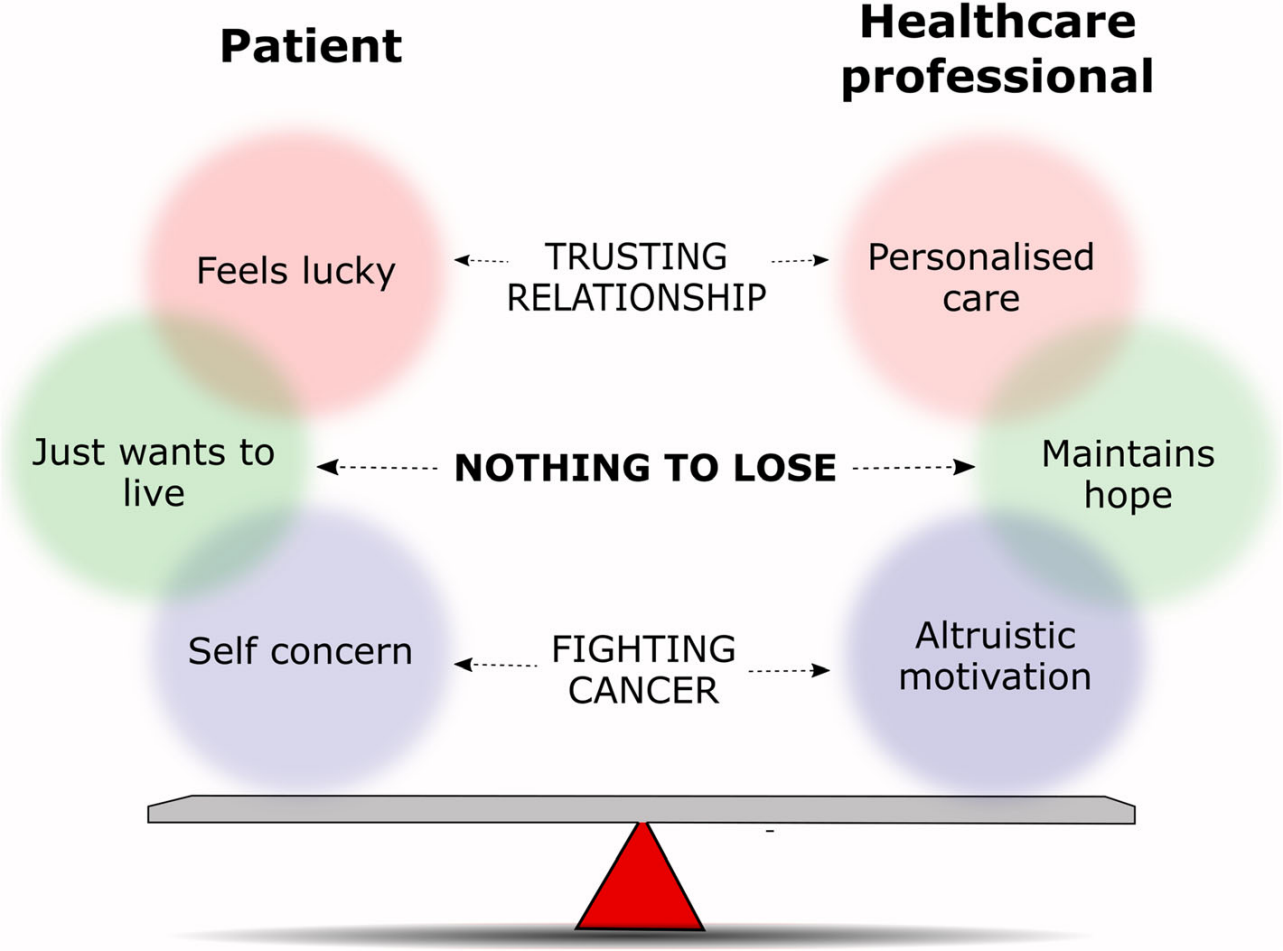
Data categories and subcategories. Legend: Visual representation of the categories and subcategories identified during analysis of transcripts from both patients and healthcare professionals

Nothing to lose was identified as a core category for both the healthcare professionals involved and patients with incurable cancer (see Table 2, Fig. 1).

*‘It’s a “no brainer”, really. If a consultant says to you, “Listen, we’ve got an opportunity here to improve your situation. It hasn’t been done before. Would you be interested in doing it?” And you have nothing else. There is no other hope in the room, this is the only thing on the table - what are you going to do?* (P01)

This core category conveyed a fairly desperate situation, where time was running out and in the face of a dire prognosis, everyone – patients and professionals alike - were searching for a “life-line”. Conversely, the three patients who declined the offer of a clinical trial, did so after much deliberation. They felt they had ‘nothing to lose’ by saying ‘no’ to a clinical trial and instead opted for standard chemotherapy – which for them seemed a safer option.

**Table 2:** Key excerpts for identified categories and subcategories of data

Category/ subcategory	Excerpt (patient participants are italicized)
1. NOTHING TO LOSE *(core category)*	‘*I didn’t want to say too much. I told them (family) I’m going on a drug trial. But that’s where it stops. I said, “They offered me a drug trial. I have nothing to lose.” And they says, “Well that’s all right, go ahead, do what you need to do.”* (P06) *‘I’m not gonna commit to 2 years of the rest of my life to that.’* (P07 – trail decliner) ‘I think if people didn’t think, or didn’t hope that they would get life extension, no matter how well we brief them, I doubt they would actually take part in Phase I trials.’ (HcP01)
1.a Just want to live *(subcategory)*	*‘When it comes to dying or anything there’s no way, like my own view is I’m too miserable to die. I’ve too much to do and I’ve a whole lot of things more that I haven’t had any time for dying.’* (P10)
1.b Maintaining hope *(Subcategory)*	‘We ourselves are going to be hopeful that it is [the trial] going to bring some benefit. Even for Phase I studies, it is highly unlikely that you would be in a situation and caring for somebody and just thinking ...you know, you do feel that there is a possibility of individual benefit. You want that to work for them. We are delivering this in a caring therapeutic setting.’ (HcP18)
2. TRUSTING RELATIONSHIP *(Category)*	*“It’s all mind games in this disease, it feels like he is a friend in a sense because he is the one who is going to treat me for this disease … he’s the one that is working to make things better for me.”* (P01) *‘We didn’t know really whether it would be a good or bad thing. We just wanted to talk to [GP] - somebody with more experience about it.’* (P03 - trail decliner) ‘I know that it is sometimes a scary position to be in, to be the trusted doctor, especially if you have been looking after them for a number of years, they will do anything you ask them.’ (HcP10)
2.a Feel lucky *(Subcategory)*	*‘She* [clinical research nurse] *says, “You will be coming up more often”, which is what I want. I want to get a bit more attention. “You’ll be treated like a VIP” she says. I went, “That’s fine.”* (P06)
2.b Personalised Care *(Subcategory)*	‘My view is that the best quality care that I can provide for a patient is through consideration for clinical trials. They drive high quality care. They may not provide the answer but in the process of doing it, they provide a very strong methodological framework to carry out high quality clinical care. So my commitment to patients is to carry out the best care I possibly can.’ (HcP06)
3. FIGHTING CANCER *(Category)*	*‘Now it’s just a matter of ‘here we go again’. It’s just the circle of life. It’s part of my life and cancer is a big part of my life. But dying is not part of my life... So, fighting the cancer is just what I have to do.’* (P15) “These patients would grab anything to fight this disease.” (HcP02)
3a.Self concern *(Subcategory)*	*‘Well I am being totally selfish in my trial... Because the part of the trial that might help people in the future, I don’t really care at this stage, I just want to live myself. I’m not in a situation to start thinking about other people’s cures in the future. I need my own treatment now.’* (P14)
3b. Altruistic Motivation *(Subcategory)*	‘Most people still ultimately – they may tell themselves they are only doing it for altruistic reasons, but I think ultimately most people are doing it because they think it is the best thing for them. But the altruism still weighs very, very highly for them. I think there are a few individuals who just say, “Well I’ve got two options. I can have treatment, or I can have treatment which is going to help other people more, so I am going to do that.” (HcP06)

Table legend: P = patient; HcP = healthcare professional. Patient participant responses are italicized

The core category of ‘nothing to lose’ transcended all other data within the study, whilst the grounded theory analysis confirmed that it was connected to (and often under-pinned) the other categories and subcategories that will be presented below.

#### Subcategory 1.a - I just want to live (patients)

For patients, being told that their cancer was now incurable was a critical juncture in their illness experience and they all struggled to come to terms with their prognosis. In this acute situation, patients were determined not to give up (HcP13):

*‘They no longer think they can get rid of my cancer anymore. If it works really well, the chemo combined with the trial drug, can make the cancer go away, can shrink it significantly, can shrink it a little bit, or can stop it growing. And I would like the best of those options-to make it go away.’* (P14)

Incongruous perceptions were apparent, in that individual patients were aware that their cancer was incurable, but still hoped that the trial drugs might provide a cure. The concept of ‘hope’ was recurrent and a key ‘driver’ for patients to do something to extend life. It is evident that the power of their hope - or perhaps their desperation - is such that they ‘cling-on’ to any possibility that their cancer can “go away”.

#### Subcategory 1.b - Maintain hope (healthcare professionals)

The analysis outlined in Table 2, section 1.b confirms the importance professionals placed on maintaining hope. The option of a clinical trial was described as an important therapeutic mechanism by which to maintain hope.

*‘I think if people didn’t think, or didn’t hope that they would get life extension, no matter how well we brief them, I doubt they would actually take part in Phase I trials.’* (HcP01)

There was consensus amongst professionals, that this group of patients would ‘*grab anything’* (HcP02), because ‘*there is nothing else and that is the long and short of it.*’ (HcP13). In this situation they didn’t want to ‘dash hope’, accepted a ‘degree of false hope’ on the patients’ behalf and always sought to have ‘something else to offer’, even as they conveyed bad news.

### Category 2 - TRUSTING RELATIONSHIP

Building on the data in section 1, all participants in this study reported there was a very positive and trusting relationship between patient and clinician. In the case of trial decliners, their trust was with their GPs, whilst those patients that accepted the offer of a trial had greater trust in their oncologist.

*‘I would have gone on the trial anyway, cos of the trust I have in Dr S already. If he said, “Go get chemo with us* [standard treatment]*” or, “Chemo on the trial is better than chemo outside the trial.” I would have said, “Of course.”’* (P14)

Patients looked to their oncologist for guidance and described having ‘*absolute trust’* (P01) and ‘*implicit trust’* (P02) in their doctor. Some also expressed feelings of friendship with their Oncologist (see P01, Table 2, section 2). The healthcare professionals were aware of the trust that patients placed in them and were sometimes daunted by that responsibility: *‘they will do anything you ask of them’* (HcP10, Table 2).

#### Subcategory 2.a - Feel lucky (patients)

In the context of this trusting relationship, patients described themselves as feeling lucky because they had been selected to participate in a clinical trial.

*‘But you are also being given this chance. So, you’re selected and you’re (thinking) - I’m probably the only person in Northern Ireland getting this drug at the moment. It makes me feel very lucky.’ (P14)*

This excitement seemed to centre around the fact that they were going to receive a ‘new and expensive’ drug. Many patients in the study made their decision to join a trial prior to receiving detailed trial information, indicating their decision was an instinctive response – which may have been facilitated by the trusting relationship already described.

#### Subcategory 2b - Personalised care (healthcare professionals)

In terms of the healthcare professionals, most disclosed a belief that patients would have more personalised care within a clinical trial.

*‘We would love to think that patients on standard management get optimal management all the time, but they don’t. Research participants have got the research team following them up and all the things that are supposed to happen – do. So, if you like, they have got optimal care.’ (HcP16)*

Even randomization to the standard care arm of the trial was perceived positively. There was general agreement from healthcare professionals that being in a clinical trial optimises the care experience. Patients also believed that they were getting increased personalised care as part of the clinical trial, though more frequent hospital visits were perceived negatively by trail decliners.

### Category 3 - FIGHTING CANCER

Patients who had been living with cancer for many years may have exhausted all standard chemotherapy regimes and therefore a clinical trial may be their only way of receiving chemotherapy:

*‘Now it’s just a matter of “here we go again”. It’s just the circle of life. It’s part of my life and cancer is a big part of my life. But dying is not part of my life... So, fighting the cancer is just what I have to do.’* (P15)

From the data, it seemed that on occasion patients would have considered anything that was offered. Both patients and professionals described themselves as being in a battle against the mutual enemy - cancer. Healthcare professionals understood this was a life and death situation for the patients and therefore always wanted to ‘have something else to offer them’ (HcP11).

#### Subcategory 3a - Self Concern (patients)

Patients’ reasons for taking part in a clinical trial were primarily centered on their personal desire to live longer:

*‘Well I am being totally selfish in my trial … being part of the trial might help people in the future, but I don’t really care at this stage, I just want to live myself. I’m not in a situation to start thinking about other people’s cures in the future. I need my own treatment now.’* (P14)

This excerpt exemplifies the strong desire that these patients have to continue living for as long as possible. There was no sense in the patient data that their decision was altruistic - in fact, most presented as very single-minded and the motivation was exclusively focused around their personal benefit.

#### Subcategory 3.b - Altruistic motivation (healthcare professionals)

In this theme there was a notable difference in the data between healthcare professionals and patients, whereby professionals regarded altruism as a more important motivating factor than patients did:

*‘ … And so I think for many people that is a very big driver … it’s actually the driver of helping us to learn more to help other people.’* (HcP06)

It is interesting that this is the only subcategory where data from the patients and healthcare professionals are at odds with each other. It is unclear from the data provided in this study why there was a discrepancy between patient and professional on this particular aspect.

## Discussion

This analysis confirms ‘nothing to lose’ as the substantive theory that explained patients’ and healthcare professionals decision-making regarding clinical trial participation. This was linked to two core categories – the ‘trusting relationship’ between patient and professional who were united in ‘fighting cancer’. Consistent with other study findings [6], both patients and professionals viewed the offer to participate in a clinical trial as the ‘only hope in the room’. For some patients, it is hope, and continuing to believe that a cure is possible, that helps to bring some normality to their situation and enables them to cope with a bleak prognosis [35]. From a theoretical perspective [36] hope can be generalised, such as a faith in the future, or particularised; which has a direction towards a goal. Applying this theory to our study findings, patients who ‘just want to live’ may have unrealistic and particularised hope.

Previously identified challenges with having prognostic and palliative care conversations in this clinical context [37] are reflected in our study findings. In particular, the healthcare professionals’ fear of taking hope away is highly relevant [38]. However, in relation to decision making for trial participation, this raises ethical concerns. Our findings add to the call from others [39] to focus on the potential for broader positive benefits that trial participation may bring, while not delivering particular hopes which may lead to unrealistic expectations.

The trusting relationship between patient and professional meant that all consenting patients felt their doctor saw the clinical trial as the best treatment for them as an individual. This finding is consistent with other studies [11, 25, 40] which have identified trust as a central component to trial decision-making. Findings in our study clearly showed that doctors were aware that patients trusted them and felt burdened by this, though were perhaps not aware of the power they had to influence patients’ decision making. Professionals also believed that patients on clinical trials would receive better care and more attention. This raises the question of what, or perhaps more importantly who, defines “better care”? There is the possibility that not participating in clinical trials is actually the best care option for a patient, as supportive and other palliative care measures could potentially offer a better quality of life [41], but in this study the focus of both the patient and professional was on 'fighting cancer', rather than considering palliative options.

Interestingly, the only time findings conflicted between patients and professionals in this study was patients’ prioritisation of self-concern, versus the professional’s belief that patients were motivated by altruism. Other studies have reported altruism as a factor influencing the decision to participate in a clinical trial [42], however we found none that report this dichotomy. Given their limited treatment options and poor prognosis, patients may perceive themselves as lacking the luxury of altruism when making treatment decisions in this context.

The theoretical model created through grounded theory analysis of these data is detailed in Fig. 1. As described, this model is centered around the core category of ‘nothing to lose’ which is connected to and often underpins the categories of ‘trusting relationship’ and ‘fighting cancer’, as well as all subcategories. Patient and healthcare professionals each have their own distinct subcategory within ‘nothing to lose’, ‘trusting relationship’ and ‘fighting cancer’, which are often related to each other but show key differences in viewpoints between the two groups. For example, subcategories within ‘nothing to lose’ reflect patients desire to live and their willingness to accept trial opportunities; as well as the responsibility felt by healthcare professionals to maintain hope whilst also managing patient expectations. As detailed, ‘nothing to lose’ impacted all other categories, helping to foster a ‘trusting relationship’ with healthcare professionals who were offering a lifeline in the form of a trial place; as well as fuelling patient desire to fight cancer. For ‘nothing to lose’ and ‘trusting relationship’ the patient and healthcare professional subcategories were clearly related, with each agreeing on the underlying theme, though differing in their interpretation. Conversely, for ‘fighting cancer’ subcategories were clearly opposed, with healthcare professionals falsely believing altruism to be a motivating factor for patients in trial participation. This novel model may be applicable for many cancer patients, given the range of diagnoses included in the sample - though further work is needed to confirm this. Furthermore, this model may have relevance to trial patients suffering from other diseases, such as end stage renal disease and heart failure, though again further research is needed to explore this.

This study offers a holistic perspective on decision-making by including both patients and professionals. To maintain rigour all interviews were conducted by the same author, with the support of a more experienced team who read transcripts and critiqued their content for quality assurance purposes. Use of a grounded theory approach supports this, as discourse develops freely with limited influence from the interviewer. Furthermore, given the dearth of related research this study is particularly suited to a grounded theory approach [32]. Three members of the research team contributed to the coding of the data, helping to ensure emerging themes were fully explored and to enhance reflexivity during data analysis. Furthermore, data collection was only concluded once all members of the research team agreed that data saturation had been achieved. Despite its strengths, it should be noted that this study represents a small sample from one cancer centre in the UK, and therefore the transferability of these findings to other settings is not assured. Furthermore, only 3/16 patients refused participation in the original clinical trial – which may mean the views of trial decliners are under-represented. However, this was difficult to control for and may be representative of this highly motivated study population. This sample population of patients was also predominantly male (11/16), and whilst this did not appear to obviously impact results, future work would benefit from a more equal gender split. It should also be noted that participants were not afforded the opportunity to review manuscripts for accuracy, as given the short life expectancy of patients, it was deemed inappropriate to contact them after interview. As such, healthcare professionals were also not afforded this opportunity. Finally, the present study could have benefited from the inclusion of caregivers, as this would have given a clearer picture of their involvement in the decision-making process and its impact on their lives. Further research in this area should therefore include caregivers, whilst additional international research is necessary to confirm and develop findings of the present study.

The present work has demonstrated that life limited patients are, for the most part, driven by their hope to extend life and determined to avail of any treatment opportunity. Whilst not surprising, the strength of patient hope is an important consideration for future work in this field. Researchers and healthcare professionals alike should be cognisant of the vulnerability of patients and their determination to ‘fight’ their illness, as this appears to result in patients accepting any opportunity – perhaps before fully understanding what they are agreeing to. As such, extra time and care should be taken during the consent process to explain, in detail, any potential trial outcomes and their likelihood. Based on the discussions with healthcare professionals detailed in the present study, there is a balancing act to such discussions with; a fine line between dashing and maintaining hope. However, given the vulnerability of this population it is crucial, both from an ethical and moral standpoint, that patients are fully informed of all available treatment plans and not pressured to accept any particular option. In future, use of the ‘fair transaction’ model of consent, which focuses on the risk-benefit ratio of a trial, may be more applicable for this population than the ‘autonomous authorisation’ model which is used currently [43].

## Conclusion

This study presents a valuable insight into the complex, emotionally-charged and ethically contentious situation of consent in clinical trials that have non-curative intent. It has identified the main concern of both patients and professionals as being ‘nothing to lose’ and provided insight into the important contextual issues that both parties share in relation to ‘fighting cancer’ and their ‘trusting relationship’. Alongside these sub-core categories, patients and professionals had distinct perspectives that were generally aligned to a large extent. These data clarify and add to the existing international knowledge base on this multifaceted issue, emphasising the need for further research to expand our understanding of this complex situation. Furthermore, this work highlights the responsibility healthcare professionals have in ensuring patients are fully informed regarding trial requirements and potential outcomes – a task which is both more difficult and more important considering the vulnerability of this population.

## Data Availability

The dataset analysed during the current study is not publicly available given that data was collected from one clinical setting, meaning there is potential for professionals to be identified based on the nature of their comments. As such, we do not have ethical approval to upload full transcripts. However, given the extensive excerpts used we feel there is appropriate information on this data in the public domain. Further information is available from the corresponding author on request.

## Abbreviations

UK: United Kingdom
P: Patient
HcP: Healthcare Professional
